# Heterogeneous effects of individual high-fat diet compositions on phenotype, metabolic outcome, and hepatic proteome signature in BL/6 male mice

**DOI:** 10.1186/s12986-023-00729-0

**Published:** 2023-02-08

**Authors:** Ruth Janoschek, Marion Handwerk, Eva Hucklenbruch-Rother, Lisa Schmitz, Inga Bae-Gartz, Philipp Kasper, Jan-Wilm Lackmann, Tobias Kretschmer, Christina Vohlen, Andrea Mesaros, Martin Purrio, Alexander Quaas, Jörg Dötsch, Sarah Appel

**Affiliations:** 1 Department of Pediatrics and Adolescent Medicine, Faculty of Medicine and University Hospital Cologne, University of Cologne, 50937 Cologne, Germany; 2 Cologne Excellence Cluster on Cellular Stress Responses in Ageing-Associated Diseases (CECAD), University of Cologne, 50931 Cologne, Germany; 3 Phenotyping Core Facility, Max-Planck Institute for Biology of Aging, University of Cologne, 50931 Cologne, Germany; 4 Clinic for Gastroenterology and Hepatology, Faculty of Medicine and University Hospital Cologne, University of Cologne, 50937 Cologne, Germany; 5 Institute of Pathology, Faculty of Medicine and University Hospital Cologne, University of Cologne, 50937 Cologne, Germany

**Keywords:** Diet-induced obesity, Differential dietary compositions, Phenotypic outcome, Liver proteome analysis

## Abstract

The multitude of obesogenic diets used in rodent studies can hardly be overviewed. Since standardization is missing and assuming that individual compositions provoke individual effects, the choice of quality, quantity and combination of diet ingredients seems to be crucial for the outcome and interpretation of obesity studies. Therefore, the present study was conducted to compare the individual effects of three commonly used obesogenic diets, mainly differing in sugar and fat content. Besides basic phenotypic and metabolic characterization, one main aspect was a comparative liver proteome analysis. As expected, the obtained results picture differentiated consequences mainly depending on fat source and/or fat- and sugar quantity. By confirming the general presumption that the choice of nutritional composition is a pivotal factor, the present findings demonstrate that a conscious selection is indispensable for obtaining reliable and sound results in obesity research. In conclusion, we strongly recommend a careful selection of the appropriate diet in advance of a new experiment, taking into account the specific research question.

**Supplementary Information:**

The online version contains supplementary material available at 10.1186/s12986-023-00729-0.

## Background

Research worldwide is targeting the steadily rising prevalence of obesity and the resulting associated health issues of future decades. Commonly rodents, and in particular mouse models, are used to investigate the underlying mechanisms and consequences of overweight and obesity on metabolism in vivo [16]. Besides genetically obese mouse models like the leptin (ob/ob) or leptin receptor (db/db) deficient mice, obesogenic diets are the main tool to generate overweight and obese mice.

Currently offered obesogenic diets are termed high-fat-diet (HFD), diet induced obesity (DIO), cafeteria-diet or western-style diet (WSD) [27]. However, diets are lacking standardization and the nutritional compositions of the respective diets differ quite strongly depending on manufacturer or research lab. Especially the amounts of calories from lipids and/or carbohydrates deviate significantly from one another [13]. Consequently, each diet provokes a unique obese phenotype, depending, on i) the respective mouse strain, ii) age, iii) sex and iv) feeding period, resulting in a substantial comparability dilemma.

In the past decades, a so called high-fat-diet (HFD), providing 60% of calories from fat was one of the most commonly used obesogenic diets [2, 7, 36, 47]. Reflecting contemporary eating habits of our western population, the question arises if this diet, predominantly comprised of lard, is the appropriate choice for mimicking the most prevalent cause of overweight and obesity in humans. Usually, the majority of people in western societies consuming unhealthy diets prefers fat- and sugar-rich foods because of its palatability [24]. Hence, an adjustment of experimental diets and a more precise imitation of human nutrition habits might result in more reliable findings in obesity research. Unfortunately, a large number of special diets is commercially available by now. This leads to a complex mass of data, and the evaluation and comparability of overall research results has become a major challenge [8]. Therefore, the aim of the present study was to compare the effects of three different commercially available and commonly used obesogenic diets on rodents to assess the range and extent of differing effects.

In this study the following diets were used: firstly, a common but rather artificial HFD providing 60% calories from fat (mainly lard) [2, 23, 35, 47]. Secondly, a Western Style-diet (WSD) providing 43% metabolizable energy from fat (21% butter fat) plus very high amounts (34%) of sugar (sucrose), resembling the commonly used ‘RD Western Diet’ D12079B (Research Diets inc.). This diet is used in research of obesity, the metabolic syndrome, diabetes, hypertension or atherosclerosis [15, 30, 37, 42]. Thirdly, one diet out of the wide DIO (diet-induced obesity) series was chosen, a locally produced DIO, providing 45% of calories from fat (mainly lard) and high amounts (21%) of sugar (sucrose) as well. The DIO-diet corresponds to the frequently used D12451 (Research Diets inc.), a diet designed to induce obesity, metabolic syndrome and type II diabetes [34, 42]. All experimental diets were compared to a regular maintenance chow (standard diet, SD) with 9% metabolizable energy from fat and low sugar content.

All three different common obesogenic diets were compared with regard to their effects on phenotype, glucose homeostasis, adipose tissue morphology and liver metabolism and proteome.

In addition, the data presented might assist in selecting an obesogenic diet appropriate for the particular research question when designing future experiments.

## Methods

**Fig. 1:**
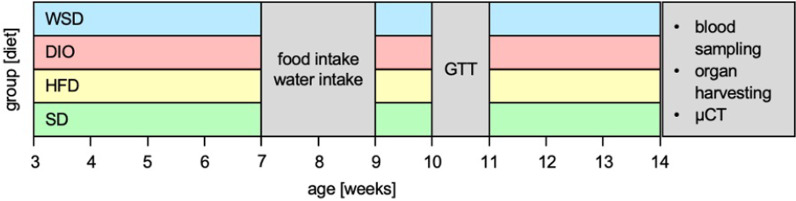
Experimental design, four groups of mice feeding different diets were monitored over a period of eleven weeks post weaning. Food & water intake were recorded during postnatal week 7 and 8, and intraperitoneal glucose tolerance test was performed in week 10. In postnatal week 14 animals were euthanized

*Mice* The study was approved by the appropriate governmental authority (AZ 84–02.04.2016.A046, Landesamt für Natur, Umwelt und Verbraucherschutz Nordrhein-Westfalen, Germany). For systematic heterogenization, the animal experiments were conducted in two consecutive passages. In total, 72 C57BL/6N male mice (Janvier, France), three weeks of age, were randomly split into four groups upon arrival, in total 18 animals per group. Each group received one of the four scheduled diets until the end of the experiment at postnatal week 14 (Fig. 1). Groups were labeled according to their respective diets: SD: standard chow diet; HFD: high-fat-diet; DIO: diet-induced obesity; WSD: western-style diet (overview Table 1, further details see Additional file 1: Table AT1). Food and water were available ad libitum and were only withdrawn if experimentally necessary. All animals were housed in groups (max. 3 animals/cage) and were maintained at 22 °C on a 12-h light, 12-h dark cycle. Body weight (BW) was determined weekly.

**Table 1:** dietary overview, for further specifications see Additional file 1: Table AT1

	SD	HFD	DIO	WSD
	Complete feed for rats & mice	C1057, modified	DIO-45 kJ% fat (Lard)	Western-Diet butter fat
Company	Ssniff	Altromin	Ssniff	Ssniff
Order number	V1534-R/M-Maintenance	10005791	E15744-344	E15775-34
Additional information	Complete feed for rats & mice	High fat diet	HF diet for rodents with lard (& soybean oil) corresponds to D12451 Research Diets	HF / High cholesterol diet for mice & 1% corn oil
Metab. energy [kcal/kg]	3225,0	5237,0	4615	4595
Metab. energy [MJ/kg]	13,5	21,9	19,3	19,2
Fat [kJ%]	9,0	60,0	45,0	43,0
Protein [kJ%]	33,0	16,0	20,0	15,0
Carbohydrates [kJ%]	58,0	24,0	35,0	42,0
Fat [g/kg]	33,0	351,0	236,0	220,0
Protein [g/kg]	190,0	208,0	220,0	173,0
Sugar [g/kg]	47,0	121,0	211,0	344,0

*Food & water intake* food and water intake were quantified at the 7th and 8th week of age using special nipple potions and food containers. During the assessment of food consumption, mice remained in their individual groups of 2–3 animals per cage for social and animal welfare reasons (e.g., avoiding the stress of single housing during and after the procedure). Quantified amounts of consumed food/water per cage were divided by the number of animals and objected to further calculations.

*Glucose tolerance tests* at the age of 10 weeks, intraperitoneal glucose tolerance tests (ipGTT) were performed as described before [10]. Briefly, after a fasting period of 6 h at the beginning of light phase, each animal received an i.p. injection of 2 g glucose/kg body weight. Blood glucose was determined prior to injection (0) and 15, 30, and 60 min (min) after injection.

*Organ harvesting* at 14 weeks of age (99 ± 2 days), 12 animals per group were euthanized via CO_2_-inhalation, body weights were detected, blood samples were collected and transcardial perfusion was performed using 0.9% sodium chloride (Fresenius Kabi Deutschland GmbH). Body length (nose to tail root) was assessed by vertically pinning the mouse on scale paper. Body mass index (BMI) was calculated dividing body weight (gram, g) by body length (cm^2^). Adipose tissue and liver were harvested and cut into halves. One part of each tissue was incubated in 4% paraformaldehyde (PFA, 24 h; Roti®Histofix, Carl Roth, Germany) followed by paraffin embedding processes. The remaining parts were snap frozen and stored at − 80 °C until further processing. A subset of 5 animals per group was euthanized via cervical dislocation and stored intactly for micro computed tomography (µCT) analyses at − 20 °C.

*Quantification of body fat by μCT* was performed as described previously [38]. Briefly, whole mice were scanned post mortem with a μCT scanner (SkyScan 1176, Bruker, Belgium) with an isotropic voxel size of 35.26 μm^3^. The x-ray settings for each scan were 45 kV and 475 μA using a 0.5 mm aluminum filter. All scans were performed over 360° with a rotation step of 0.6° and a frame averaging of 2. Images were reconstructed, analyzed and visualized using the NRecon, CTAn and CTVox software, respectively (Bruker, Belgium). Images were segmented based on tissue density for both total volume and fat volume. Total fat volume was further segmented into visceral and subcutaneous fat using the abdominal muscular wall as orientation.

*Histological analysis of adipose- and hepatic tissue* 5 µm (µm) sections of epigonadal white adipose tissue (WAT) were prepared from paraffin embedded tissue samples. Slices were stained with hematoxylin and eosin (H&E) for microscopic examination. In total, 10 independent pictures per object slide containing 3–5 WAT consecutive slices were captured with a 40 × magnification (via Olympus BX43F with CellSens Dimension software (DP80 dual CCD Camera, cellSens Dimension (V1.8)). Subsequently, blinded images were analyzed via Adiposoft (Fiji, ImageJ), manual mode with 0.1613 µm/pixel, minimum diameter 10 µm and maximum diameter 100 µm, excluding cells on edges.

Liver samples were processed to 3 µm sections, H&E stainings were performed and object slides were analyzed as described previously [5, 25]. Briefly, the degree of steatosis, inflammation and hepatocellular ballooning were evaluated by an expert in liver pathology blinded to the dietary conditions.

*Analytical procedures* Serum levels of leptin were measured by Enzyme-Linked Immunosorbent Assay (ELISA) according to the manufacturer's guidelines (mouse leptin ELISA (EZML-82 K), Millipore CorpBillerica, MA). Liver function tests (aspartate-aminotransferase (AST) alanine-aminotransferase (ALT), triglycerides, cholesterol and high-density lipoprotein (HDL)-cholesterol) were quantified by the institute for clinical chemistry of the University Hospital of Cologne according to standard procedures.

*Proteomics (liver tissue)* Liver samples were homogenized using a ball mill on dry ice (Precellys Evolution, settings: 2 × 20 s with 15 s break, 5800 rpm) in 500 µl 6 M guanidinium hydrochloride. 50 µg of proteins for each sample were reduced and alkylated followed by trypsin digestion. Peptides were purified using styrenedivinylbenzene reverse phase (SDB-RP) stage tips and stored at 4 °C prior analysis. All samples were analyzed by the CECAD Proteomics Facility. Samples were measured on a Q Exactive Plus Orbitrap mass spectrometer that was coupled to an EASY nLC (both Thermo Scientific). Peptides were loaded with solvent A (0.1% formic acid in water) onto an in-house packed analytical column (50–75 µm I.D., filled with 2.7 µm Poroshell EC120 C18, Agilent). Peptides were chromatographically separated at a constant flow rate of 250 nanoliters per minute (nL/min) using the following gradient: 3–5% solvent B (0.1% formic acid in 80% acetonitrile) within 1.0 min, 5–30% solvent B within 121.0 min, 30–40% solvent B within 19.0 min, 40–95% solvent B within 1.0 min, followed by washing and column equilibration. The mass spectrometer was operated in data-dependent acquisition mode.

The mass spectrometry (MS) 1 survey scan was acquired from 300 to 1750 mass-to-charge ratio (m/z) at a resolution of 70,000. The top 10 most abundant peptides were isolated within a 1.8 Thomson (Th) window and subjected to HCD fragmentation at a normalized collision energy of 27%. The AGC target was set to 5e^5^ charges, allowing a maximum injection time of 55 ms (ms). Product ions were detected in the Orbitrap at a resolution of 17,500. Precursors were dynamically excluded for 25.0 s (s).

All mass spectrometric raw data were processed with MaxQuant (version 1.5.3.8, [12]) using default parameters. Briefly, MS2 spectra were searched against the Uniprot mouse reference proteome (UP000000589, downloaded at: 26.08.2020) database, including a list of common contaminants. False discovery rates (FDR) on protein and peptide-to-spectrum match (PSM) level were estimated by the target-decoy approach to 1% (Protein FDR) and 1% (PSM FDR) respectively. The minimal peptide length was set to 7 amino acids and carbamidomethylation at cysteine residues was considered as a fixed modification. Oxidation (M) and Acetyl (Protein N-term) were included as variable modifications. The match-between runs option was enabled. Label-free quantification (LFQ) was enabled using default settings.

Perseus (MaxQuant, version 1.6.15.0) [44] was used for comprehensive analysis of the obtained data (global proteomics analysis + principal component analysis (PCA)) and a heatmap was created with Instant Clue (cluster settings: euclidean/euclidean; v0.10.10.20210316, University of Cologne) [31]. Volcano plots were generated with GraphPad Prism 9 and FunRich 3.1.4 was used for the comparison and visualization of the significantly changed proteins [18, 32]. The mass spectrometry proteomics data have been deposited to the ProteomeXchange Consortium via the PRoteomics IDEntifications (PRIDE) [33] partner repository with the dataset identifier PXD034538.

*Statistical analysis* Data are presented as means ± standard deviation (SD). Analyses were performed with GraphPad Prism 9. Significant outliers were excluded utilizing the GraphPad internal ROUT (Robust regression and Outlier removal) method. For multiple comparison tests, experimental groups were compared to the control group. Depending on sample number and Gaussian distribution different statistical tests were used.

More specifically, longitudinal body weight was analyzed with mixed-effects analysis followed by Dunnett’s multiple comparison tests.

GTT and adipocytes diameters were calculated using a two-way Anova followed by Dunnett’s multiple comparison test. Final body weight, BMI, water intake and kidney weights were calculated with an ordinary one-way Anova (normality test passed) followed by Dunnett’s multiple comparison test**.** Absolute WAT, relative WAT, body length, total fat/volume µCT, visceral fat/volume, serum leptin, food intake, protein intake, sugar intake, fat intake, adipocytes (total area, cell count, cell size) and serum liver parameters (AST, ALT, triglycerides) were calculated using a nonparametric one-way ANOVA (Kruskal-Wallis) followed by Dunn's multiple comparisons test. Statistical significance was defined as *p* < 0.05 and indicated with *p < 0.05, **p < 0.01, ***p < 0.001 and ****p < 0.0001 within the respective graphs. The calculations were performed according to a previous agreement with the Institute of Medical Statistics, Informatics and Epidemiology, University of Cologne.

## Results

### Phenotype

**Fig. 2:**
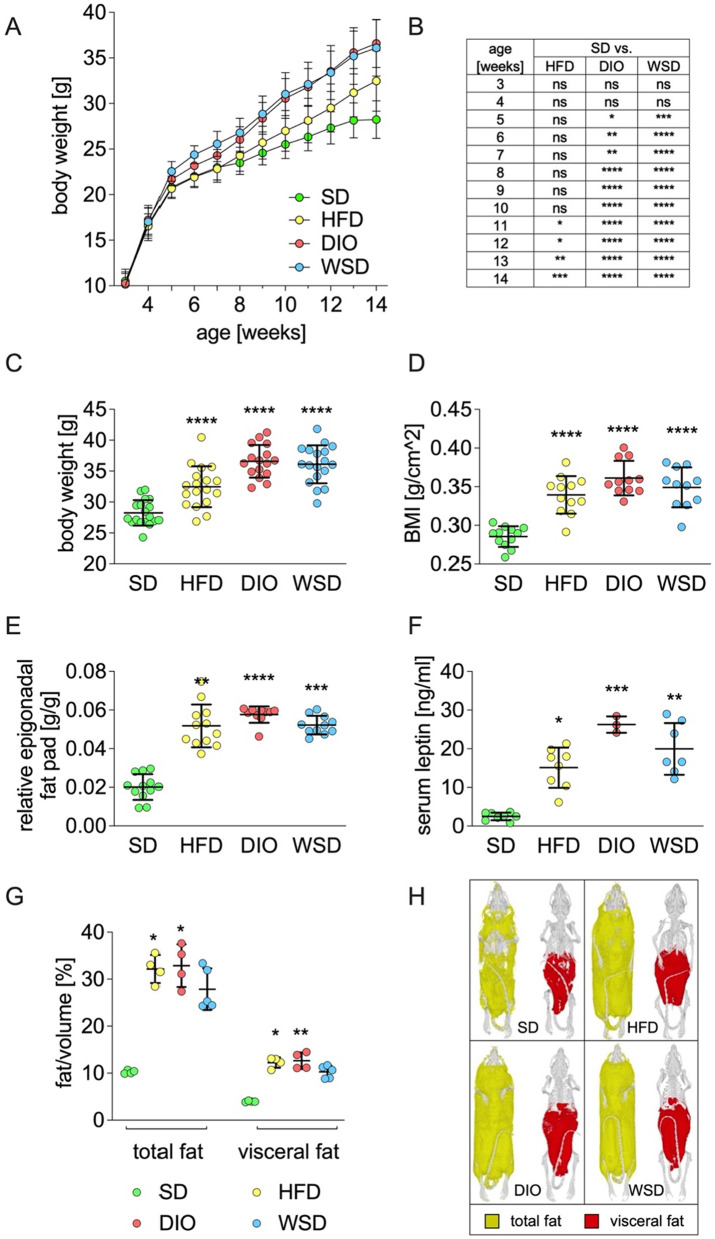
phenotypic effects after 11 weeks feeding of the experimental diets. **A** body weight; **B** body weight differences of 1A, experimental groups versus SD group; **C** final body weight; **D** body mass index (BMI); **E** epigonal fat pad weight relative to body weight; **F** serum leptin levels; **G** total and visceral body fat content (assessed by µCT analysis); **H** representative pictures of µCT analysis; n = A + C: SD, HFD, WSD: n = 18; DIO: n = 16; D + E: n = SD + HFD: 12, DIO: 10, WSD: 11; F: SD + HFD 8, DIO: 3, WSD: 7; G: SD: 5, HFD + DIO: 4, WSD: 6

During the experimental period of eleven weeks, body weight gain varied significantly between the experimental groups when compared to SD (Fig. 2A). In detail, DIO and WSD displayed significant higher body weights starting in postnatal week 5 (Fig. 2B) while HFD body weight remained similar to SD animals until postnatal week 11. At the end of the experiment, body weights of all experimental groups were significantly higher compared to SD: HFD = 115%, DIO = 129%, WSD = 127% of SD (Fig. 2C).

As body length did not differ among groups (Additional file 1: Fig. AF1A) the BMI of all experimental groups were significantly greater than SD’s BMI (Fig. 2D). These findings were accompanied by increased epigonadal fat pad weights, of which DIO animals revealed the highest amount of epigonadal fat tissue mass (Fig. 2E). Determination of random fed serum leptin levels confirmed the significant increase of adipose tissue in all experimental groups with DIO exhibiting the highest amount of serum leptin concentrations (Fig. 2F). µCT analyses of a subset of animals displayed significantly higher amounts of total- and visceral fat in the HFD and DIO group when compared to SD animals. Fat volumes of WSD animals were also elevated but did not reach statistical significance (Fig. 2G, H; all µCT-scans see Additional file 1: Fig. AF1B).

### Glucose metabolism

**Fig. 3:**
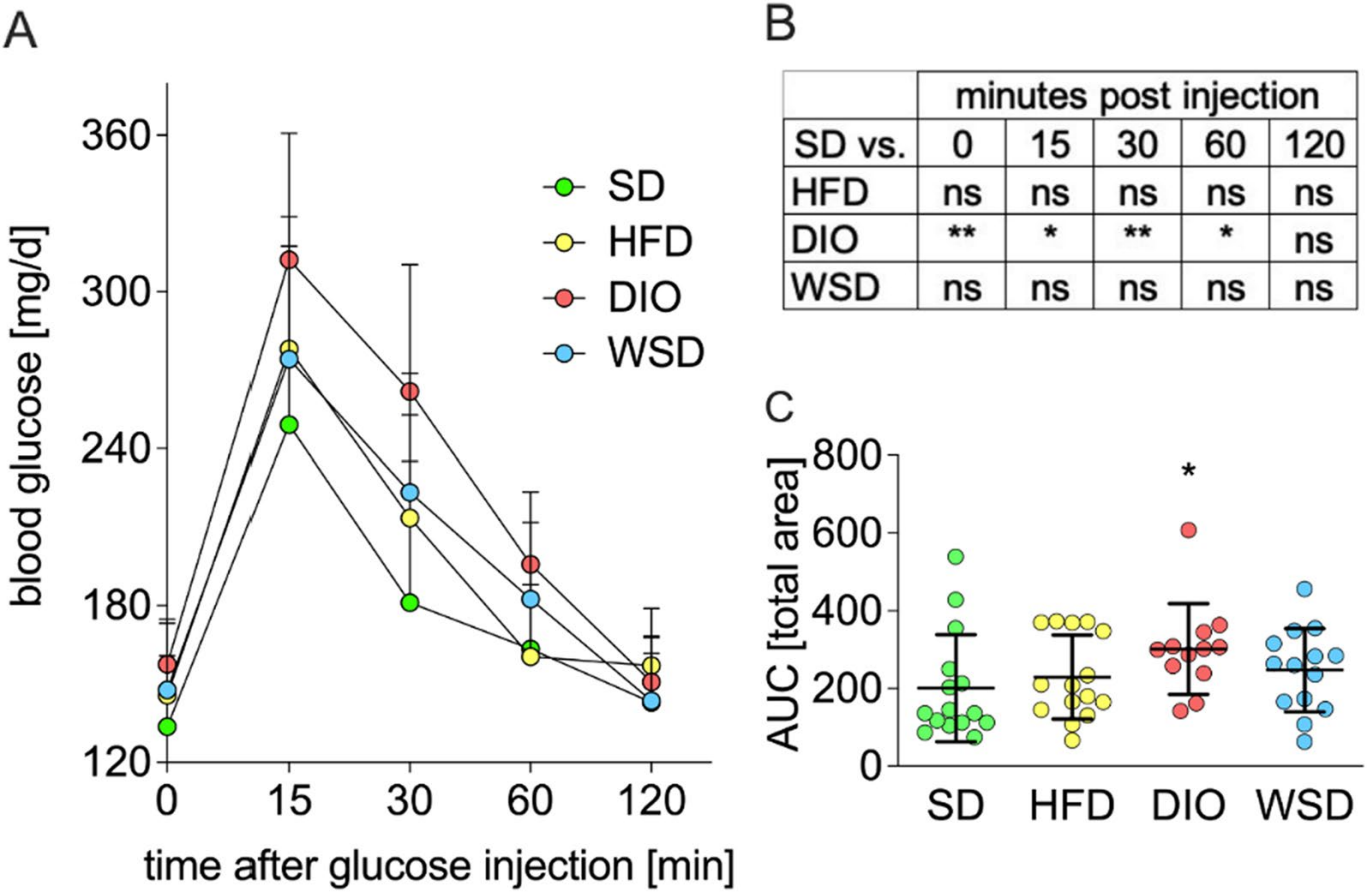
Glucose metabolism in postnatal week 10. **A** glucose tolerance test; **B** statistical differences of A with experimental groups compared to SD; **C** area under curve (AUC) analysis of A. n = SD + HFD = 15, DIO = 12, WSD = 14

In postnatal week 10, an ipGTT revealed disturbed glucose utilization in all experimental groups, but only DIO animals displayed significant differences in comparison to SD, (Fig. 3A, B). More specifically, not only fasted blood glucose was significantly elevated when compared to SD (Fig. 3A, B), but also 15, 30 and 60 min after glucose injection blood glucose levels were markedly increased in DIO animals, indicating an altered glucose tolerance (Fig. 3B). Area under curve (AUC) calculations complemented these findings (Fig. 3C).

### Food and water intake

**Fig. 4:**
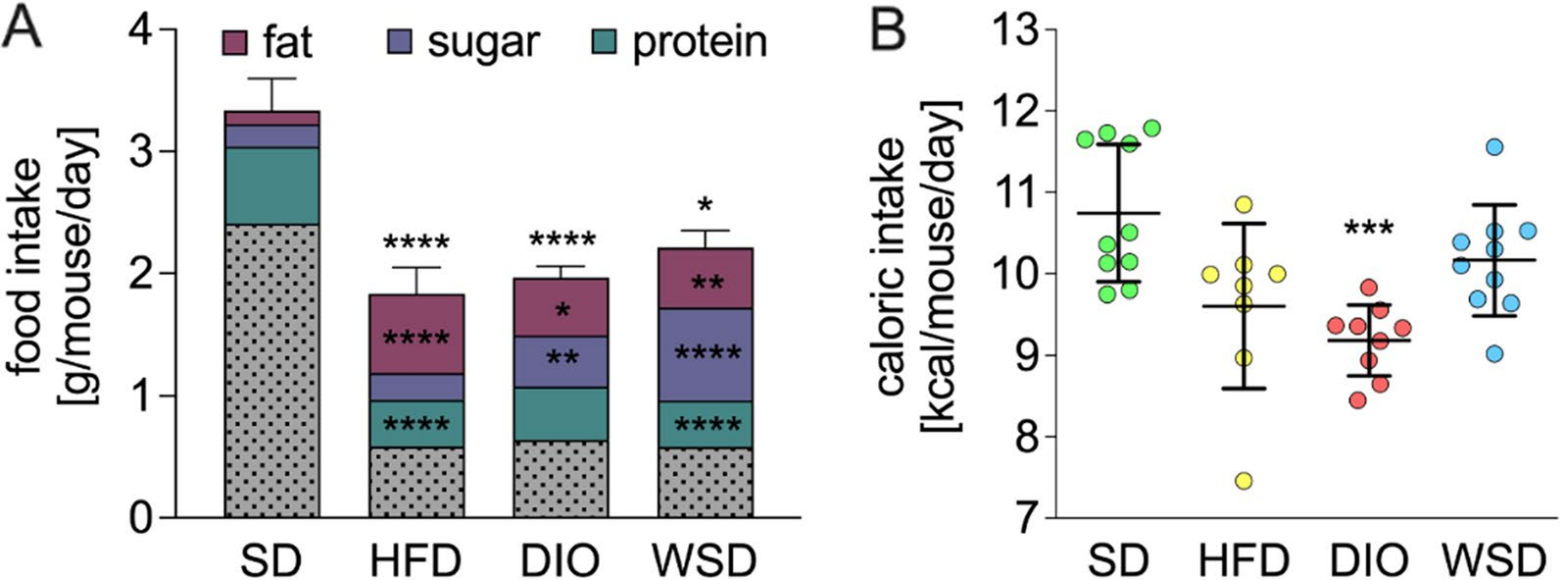
Daily food Intake during week 8–10. **A** daily food intake including daily consumption of fat (purple), sugar (blue), protein (green) and remaining ingredients (grey). Statistical differences of total food intake are indicated by asterisks above the corresponding error bars (respective experimental group versus SD group), statistical differences of the distinct ingredient are indicated in the respective colored section (experimental group vs. SD); **B** daily caloric intake; n = 8–12

The assessment of food and water intake revealed that all experimental groups consumed significantly less amounts of food when compared to SD (Fig. 4A). A more detailed analysis of dietary components presented divergent results. Protein intake was highest in the SD group, while all experimental groups exhibited a notably reduced amount of protein intake, which was even highly significant in the HFD and WSD group (Fig. 4A). As expected, fat intake was significantly elevated in all experimental groups and strongest in HFD animals (Fig. 4A) whereas sugar intake was significantly elevated in DIO and WSD (Fig. 4A). The conversion of g/mouse/day into kcal/mouse/day only partially relativized these results (Fig. 4B). Although the caloric intake of HFD and WSD turned out to be almost similar to SD, the DIO group still displayed a significant reduction in caloric intake, contrasting the clearly elevated body weights in the measurement period compared to SD (week 7–9, Fig. 2B).

Quantification of water consumption revealed a significantly lower water intake in all experimental groups compared to SD (HFD: 63,9%, DIO: 63,9% and WSD 64,1%) (Additional file 1: Fig. AF2). Because of the observed differences in protein- and water intake, kidney weights were recorded additionally, exhibiting markedly decreased kidney weights in all experimental groups (Additional file 1: Fig. AF2).

### Adipocyte histology

**Fig. 5:**
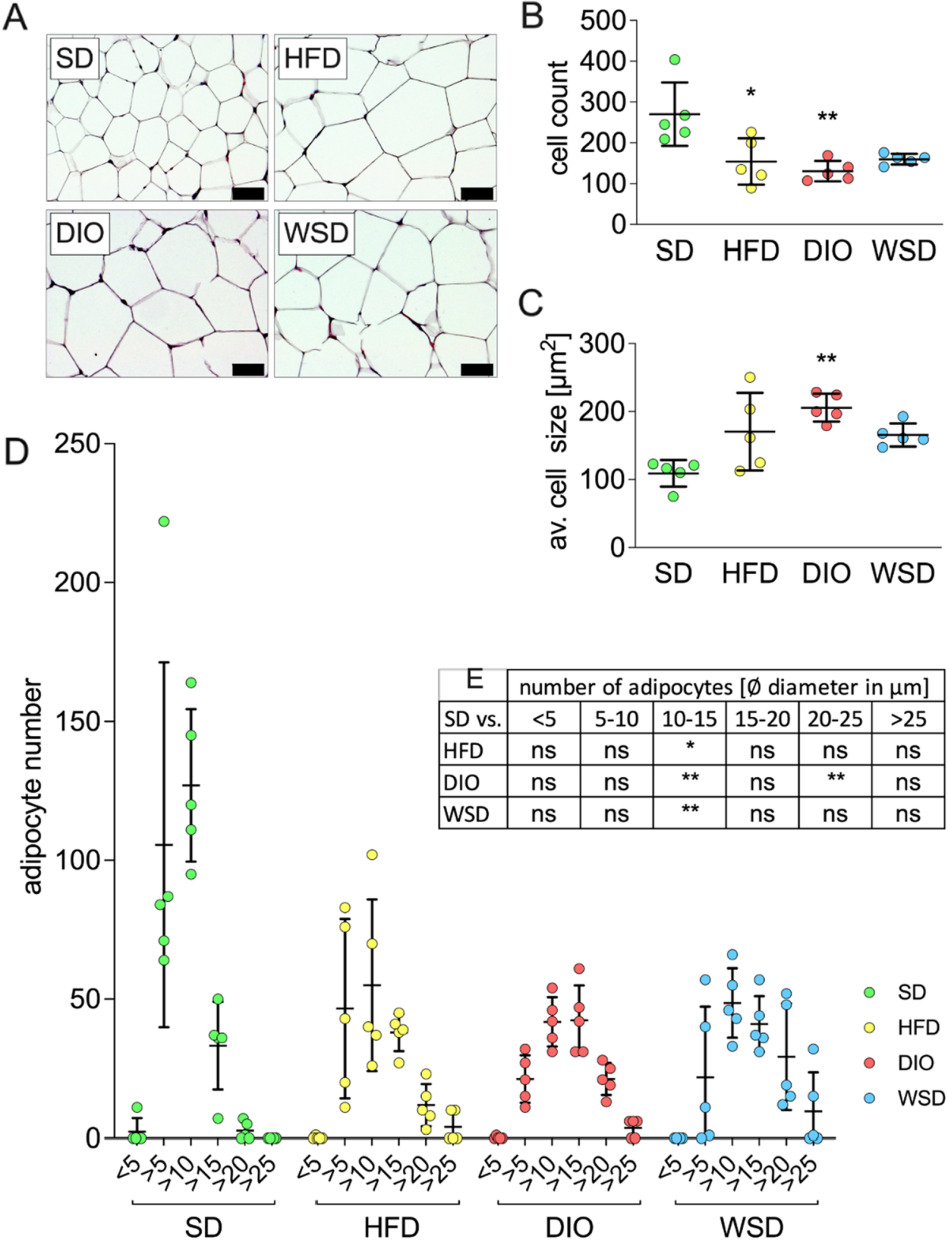
Adipocyte histology. **A** representative sections of H&E stained epigonadal white adipose tissue, scale bar: 50 µm; **B** number of counted cells per total area analyzed in the respective group; **C** average cell size; **D** distribution of adipocyte number according to diameter; **E** statistical differences of D compared to SD; n = 5 (**A**–**F**)

Histological analysis of H&E stained epigonadal white adipose tissue displayed enlarged adipocyte cells in all experimental groups when compared to SD (Fig. 5A), indicating adipocyte hypertrophy. The number of counted cells of a comparable area differed between the groups (Fig. 5B, Additional file 1: Fig. AF3). A shift in average cell diameter and cell size was detectable in the experimental groups compared to epigonaldal adipocytes of SD animals (Fig. 5C, D). Epigonadal adipose tissue of all experimental groups revealed a significantly reduced number of small cells (ø 10–15 µm) compared to SD animals, and the number of larger cells (ø 20–25 µm) was significantly elevated in the DIO group (Fig. 5E).

### Serum / liver analyses

**Fig. 6:**
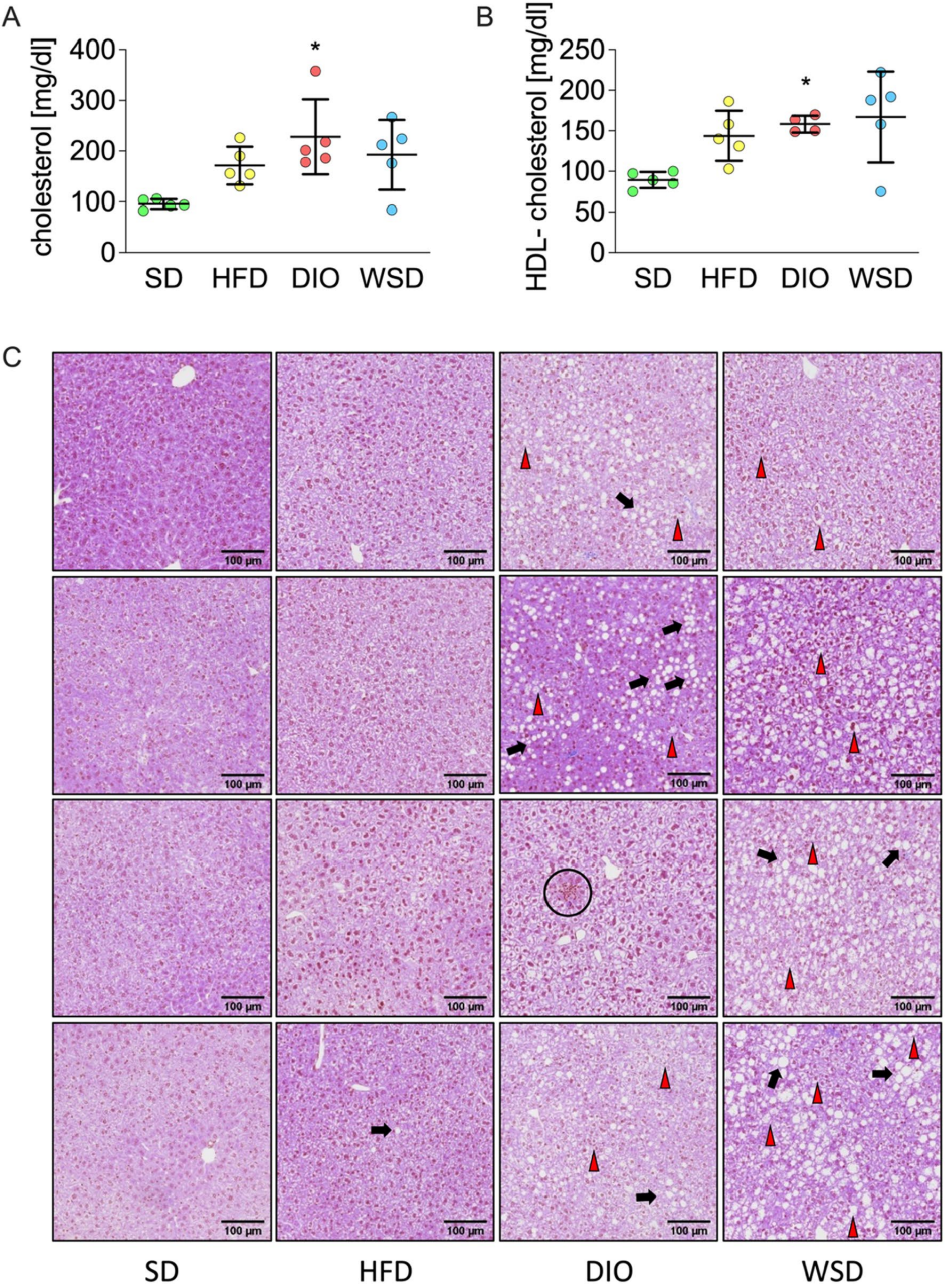
Serum liver parameters and liver histology: **A** total serum cholesterol; **B** serum HDL-cholesterol; n = 5; **C** representative sections of H&E- stained liver tissue scans, black arrows = fat deposits; red arrowheads = hydrops; black circle = inflammation; scale bar = 100 µm

Aspartate-aminotransferase (AST) and alanine-aminotransferase (ALT) are established liver function tests and markers of hepatic injury, and their levels have been shown to increase with body weight gain [43]. Therefore, serum of all groups was analyzed to assess dietary effects on liver function tests. This was accompanied by serological analysis of markers of lipid metabolism including triglycerides, total cholesterol and high-density lipoprotein (HDL) cholesterol. No significant differences in AST and ALT levels could be detected (Additional file 1: Fig. AF4). Serum triglyceride content was lowest in the WSD group while HFD and DIO exhibited a moderate increase when compared to SD animals (Additional file 1: Fig. AF4). Although total cholesterol levels were increased in the sera of all experimental groups, only the DIO group revealed a significant difference compared to the SD group (Fig. 6A). Regarding high-density lipoprotein-cholesterol (HDL-C), all experimental groups displayed a trend of elevated HDL-C levels when compared to the SD group, but only the WSD group exhibited a significant increase of HDL-C (Fig. 6B).

To evaluate the effects of the different diets on hepatic lipid content, liver histomorphology was analyzed. While HFD livers did not show particular abnormalities compared to SD, DIO and WSD livers clearly presented liver hydrops as well as hepatic lipid accumulations up to 30% (Fig. 6C and Additional file 1: Table AT1), indicating early stages of hepatic steatosis and nonalcoholic fatty liver disease (NAFLD). Within our samples, only one DIO liver exhibited inflammatory characteristics, suggesting a potential development towards a more progressive form of NAFLD namely nonalcoholic steatohepatitis (NASH) [28].

**Fig. 7:**
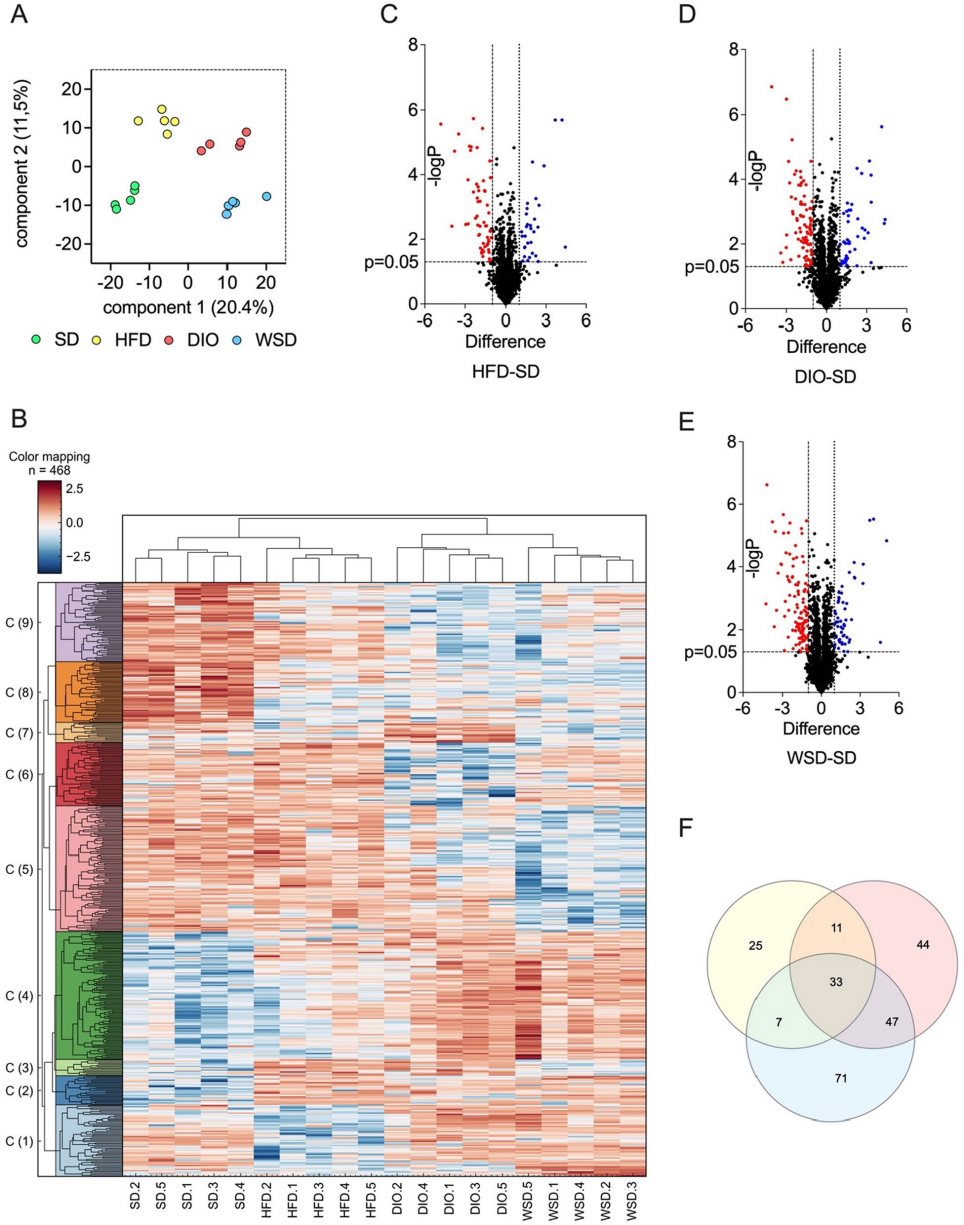
Liver proteomics analyses; **A** principal component analysis (PCA); **B** cluster analysis (cluster settings: euclidean/euclidean); **C** volcano plot HFD-SD; **D** volcano plot HDIO-SD; **E** volcano plot WSD-SD; **F** dotted vertical lines indicate a fold change of < / > 2, Venn diagram, detailed lists of overlapping proteins see ST4; **A**–**F**, n = 5 per group

To get further insights into the differential dietary effects on liver protein signatures, liver samples were subjected to proteomic analysis. Overall, a total of 2857 proteins were identified and quantified with an FDR of 0.01. 468 of them displayed significant changes between one or more experimental groups (Data are available via ProteomeXchange with identifier PXD034538). Strikingly, all groups were clustered according to their experimental diet, not only in the principal component analysis but also in a hierarchical clustering calculation (Fig. 7A, B). Notably, the heat map presented 9 individual clusters with different pattern variations between individual groups (for details see Additional file 2: Table AT3, IC cluster export). Additionally, data were displayed as volcano plots to visualize the number and dimension of significantly altered proteins thereby pointing out the diet-specific regulation patterns (Fig. 7C–E). Finally, the direct comparison of all significantly changed proteins (respective experimental group vs. control group) revealed an overall overlap of 33 proteins within the experimental groups (Fig. 7F and Additional file 3: Table AT4). Furthermore, the Venn diagram displayed a collective consensus of 47 significantly altered hepatic proteins between DIO and WSD. DIO and HFD livers shared 11 significantly altered proteins while WSD and HFD only matched 7 significantly altered proteins (Fig. 7F and Additional file 3: Table AT4). Noteworthy, the number of exclusively altered proteins of the respective intervention group clearly varied. Thus, the WSD group displayed the highest number of significantly changed proteins (71), the DIO group still exhibited 44 proteins while the HFD group presented only 25 significantly altered proteins, when compared to SD livers. Taken together, each experimental diet resulted in a distinct hepatic protein expression signature.

## Discussion

Obesity research, to a large extend, is based on animal models of diet induced obesity. Considering the huge range of diet compositions on the one hand, and the great variance of research results on the other, the present study aimed at examining the differential effects of three commonly used diets on phenotypical and metabolic parameters, adipose tissue morphology, liver function tests and the liver proteome.

Depending on literature research and due to regional availability, a local pendant of a diet frequently used in the US was chosen. This DIO with lard being the main source of fat and with 21.1% sucrose has frequently been used in research on fatty liver, inflammation, obesity and diabetes. As a second option, WSD, a high-fat/high-cholesterol diet with a similar amount of fat plus much higher amounts of sugar (34,4%) was selected, representing – as assumed—the ‘unhealthiest’ diet in the experimental setup. Originally, this WSD has been designed to induce hyperlipidemic disorders, hypercholesterolemia and atherosclerosis. The third diet, a HFD providing 60% of calories from lard but containing only moderate amounts of sugar, has been used hitherto in our experiments [2–4, 22, 36, 38]. Thus, the experimental setting comprised three different experimental diets, namely the previously used HFD as well as the selected DIO and WSD. It was deliberately decided against feeding specific low-fat control diets produced for the respective experimental diet. Instead, a regular maintenance chow was fed, representing the normal, healthy nutrition and furthermore, enabling the comparison of all experimental diets to one single standard group.

At the end of the feeding period, phenotypical results revealed a much greater impact of DIO and WSD on body weight compared to HFD. More precisely, although the high-fat diet contained the highest amounts of fat (35%), body weight gain of the HFD group proceeded less rapidly compared to DIO and WSD animals. The discrepancy between HFD and DIO animals manifested in body fat content and serum leptin levels with DIO animals presenting the highest measurements. Surprisingly, although WSD animals exhibited similar body weights as DIO animals, body fat content and serum leptin levels did not reach DIO values.

Obviously, the combination of butter fat and very high amounts of sugar displayed more contradictory results than the combination of lard and high sucrose in DIO animals. Considering a similar dietary fat content (23,6%, DIO / 22,0% WSD) but different amounts of sugar (21.1%, DIO / 34.4%, WSD) the observed phenotype must result either from the fat quality (lard (HFD & DIO) vs. butter fat (WSD)) and/or from the varying combinations of fat plus sugar. Along with the present findings, recently Guimarães et al. described the combination of high-lard/high-sugar being more efficient in inducing obesity and glucose intolerance compared to high-butter/high-sugar or high-oil/high-sugar [19]. In contrast to Guimaraes’s study, our setup also included a high-fat/normal-sugar diet (HFD) which was frequently used in previous studies [9, 17, 45].

Further examination of parameters of glucose homeostasis revealed significantly disturbed glucose utilization solely in the DIO group while HFD and WSD displayed only moderate effects. These findings are consistent with the results of Guimarães and colleagues [19] in which an increased glucose tolerance was observed only in the high-lard, high-sugar (HLHS) group while the high-butter, high-sugar group (HBHS) did not deviate from the SD group significantly. In order to determine the underlying causes it seems to be necessary to take a closer look on the individual lipid profile. The observed differences might result from differences in the fatty acid (FA) profiles of DIO and WSD. On the one hand, the percentage of unsaturated long-chain FAs is higher in the DIO compared to the WSD. On the other hand, some middle chain FAs are only present in the WSD. However, since the breakdown of the effects of the individual fatty acid combinations was not the subject of the present study further investigations are needed to decipher the actual underlying causes.

Consequently, our results lead to the speculation that great amounts of fat (pork lard, 60 kJ%) combined with moderate levels of mono- and disaccharides have a minor impact on metabolism than the combination of lard (45 kJ%) and high-sugar levels (DIO), which exerts the greatest metabolic effects in the present study.

Naturally, one would attribute body weight gain, changes in glucose tolerance and increased amounts of body fat content to a higher caloric intake presumed by the greater amounts of metabolizable energy (ME, SD: 3057; HFD: 5237; DIO: 4615 and WSD: 4595 in kcal/kg). Interestingly, total food intake (in g/mouse/day) of all three experimental diets was significantly reduced compared to SD animals. After conversion into kcal/mouse/day, the significant reduction of caloric intake was still prominent in the DIO group, thus, clearly contrasting the significantly elevated body weights and blood glucose levels of DIO animals. According to So et. al. [39] rodents are capable of a spontaneous reduction of food intake to adjust the energy intake to an isoenergetic amount. Previous studies reported a so called “protein-leverage” describing an elevated energy intake when the percentage protein content of the diet was lowered. Furthermore, diets with a very low protein proportion (but without being a “low-protein” diet by definition) led to increased lipid storage in mice [21, 41].

However, these observations were made feeding isocaloric diets with varying amounts of protein, fat and carbohydrates and are therefore not really transferable because the diets used in the present study differ in caloric content (for details see Additional file 1: Table AT1).

Another study from the same group reported that dietary fat content does not have a significant influence on food intake, whereas protein and, to a lesser extend carbohydrates, regulate food intake [40].

A detailed breakdown of consumed dietary components in the present study confirmed that the HFD provided the greatest amount of fat while sugar intake was highest in WSD animals. Interestingly, the DIO group did not stand out in any of these parameters, nevertheless exhibiting the most significant results regarding the metabolic phenotype.

As a side note—it should be reported that all experimental groups consumed 36% less water compared to SD animals, which in contrast had the highest protein intake, presumably resulting in higher SD kidney weights.

As the liver plays a key role in lipid and carbohydrate metabolism, different hepatic parameters were analyzed: Serum analysis revealed differential alterations in AST and ALT levels but without any statistical significance. Assuming ALT as a marker of liver injury [43], WSD animals displayed the highest ALT levels, followed by DIO animals when compared to the SD group. This observation was further supported by histological examination, namely, hepatic fat accumulation and hydrops were clearly detectable in the DIO and WSD group. Interestingly, HFD animals presented the lowest AST and ALT levels of all groups as well as hardly any abnormalities regarding hepatic steatosis or inflammation, thereby demonstrating that the combined effect of fat and sugar exceeds the adverse impact of fat alone. Although triglyceride and cholesterol serum levels of the HFD group were elevated compared to SD animals, only total cholesterol in the DIO group and HDL-cholesterol levels of WSD animals reached statistical significance, further underlying the detrimental composite effects.

The unbiased approach of hepatic proteome analysis provided a clear signature in PCA analysis, emphasizing the unique features of the respective diets. Actually, the PCA pattern demonstrated the greatest spatial distance between HFD and WSD while the DIO group was found to be in between. This pattern seems to reflect the distinct dietary compositions. Since HFD and DIO share lard as a common fat source while WSD contains 21% butter fat, a closer relationship of HFD and DIO appears to be plausible.

However, HFD is 1.4 times richer in fat than DIO and 1.5 times richer in fat than WSD, resulting in clear separation. Additionally, regarding the sugar content, the pattern of the DIO is situated in between HFD and WSD: 21.1% (DIO) versus 12.1% (HFD) and 34.4% (WSD), substantiating the intermediate PCA results. The SD group was mapped clearly apart from the experimental groups. These observations were complemented by the distinct group separation in hierarchical clustering analysis.

The analyses identified WSD as the diet with the highest number of exclusively and significantly altered hepatic proteins, almost 3 times more than in HFD livers (71 vs. 25) while the concordance between HFD and WSD comprised only seven proteins. In contrast, the number of altered proteins consistent between DIO and WSD was even higher than exclusively altered proteins of the DIO group (47 vs. 44). A detailed evaluation of the resulting protein tables would, however, go beyond the scope of the present report and should be addressed in a more specific analysis. Nevertheless, without going into great detail, a first glance at the most highly regulated proteins reveals some interesting aspects. For example, peroxisomal biogenesis factor (PEX) 11A content was below the limit of detection in HFD livers while it was measurable in each sample of the remaining groups. In general, members of the PEX11 family are involved in peroxisome proliferation and maintenance [26]. Peroxisomes are highly involved in lipid metabolism, especially in the ß-oxidation of long-chain fatty acids [29] and peroxisome disorders lead to hepatic malfunctions [6]. Since several studies reported that PEX11A deficiency contributes to increased triglyceride accumulation in the liver and leads to dyslipidemia and obesity in mice [11, 46], PEX11A might seem worth a closer look. Currently one can speculate that a possible reduction of PEX11A might somehow be related to a higher amount of long-chain fatty acids in the HFD but the actual reason for the absent PEX11A protein in HFD livers in the present analysis remains to be elucidated. Future studies dealing with PEX11A would be advised to consider the present data in case they are based on a diet-induced obesity model.

As a second example, Acyl-coenzyme A thioesterase (ACOT) 11 is mentioned. Similar to PEX11A, ACOT11 was not detectable in all groups. While SD and WSD samples revealed 3 resp. 5 valid values, ACOT11 was not measurable in DIO and HFD livers. ACOT11 (synonym Thioesterase superfamily member 1 (Them1)) is involved in the mitochondrial beta-oxidation pathway. It is highly expressed in brown adipose tissue [1, 20] and in smaller amounts in the liver [48]. ACOTT11 deficient mice are protected against diet-induced hepatic steatosis and exhibit an improved glucose homeostasis [48]. In contrast, when expressed exclusively in liver, ACOTT11 leads to hepatic steatosis and diminished rates of fatty acid oxidation without affecting glucose tolerance or insulin sensitivity [14]. Taking into account the present results, it can be concluded that, ACOTT11 expression is affected by the individual diet composition.

Overall, our results support previously published reports dealing with the effects of high fat diet on rodent liver proteome. Additionally, our findings sugget that a pure HFD exerts a lower impact on liver proteome compared to DIO and WSD along with a smaller overlap in hepatic protein alterations. Interestingly, although WSD displays the highest number of significantly changed hepatic proteins and a more severe damage of liver tissue, DIO has a greater impact on phenotype, glucose tolerance and body fat content.

In conclusion, the present study provides a selective outline of differing metabolic effects of three exemplary dietary compositions, all designed for the induction of diet induced obesity. The present findings do not claim to judge or recommend any obesogenic diet but rather underline the advantage of choosing the appropriate research diet for specific research questions. The choice of the experimental diet significantly affects the induced metabolic phenotype, based on the quality and individual composition of the main ingredients, namely protein, fat and sugar. Putatively small differences might exert strong effects and should not be underestimated.

Additionally, for comparison and evaluation of findings from already published studies it seems to be imperative taking account to the diet used in each individual case. Hence, it is advisable to take a closer look on diet composition before accepting depicted effects as given as the described results might appear in different lights depending on the respective proportion and quality of fat, protein and sugar.

## Supplementary Information

**Additional file 1:** Additonal Figures (AF1–AF4) and Tables (AT1, AT2).

**Additional file 2:** Additional Table (AT3).

**Additional file 3:** Additional Table (AT4).

## Data Availability

The mass spectrometry proteomics data have been deposited to the ProteomeXchange Consortium via the PRIDE [33] partner repository with the dataset identifier PXD034538.
