# Supplementary material for: Heterogeneous effects of individual high-fat diet compositions on phenotype, metabolic outcome, and hepatic proteome signature in BL/6 male mice
Source: Nutr Metab (Lond). 2023 Feb 8;20:8. doi: 10.1186/s12986-023-00729-0 (PMC9909936; doi:10.1186/s12986-023-00729-0)
Supplement: Supplementary file 1 — Additional file 1: Additonal Figures (AF1–AF4) and Tables (AT1, AT2). [file 12986_2023_729_MOESM1_ESM.docx]

Additional tables (AT) and figures (AF):

AT 1: detailed diet compositions

| \|  \| SD \| HFD \| DIO \| WSD \| \| --- \| --- \| --- \| --- \| --- \| \| **complete feed for rats & mice** \| **10005791** \| **DIO – 45 kJ% fat (Lard)** \| **Western-Diet butter fat** \| \| Company \| Ssniff \| Altromin \| Ssniff \| Ssniff \| \| Order number \| V1534 - R/M-Maintenance \| C1057, modified \| E15744-344 \| E15775-34 \| \| Metab. energy [kcal/kg] \| 3057 \| 5237 \| 4615 \| 4595 \| \| Metab. energy [MJ/kg] \| 12,8 \| 21,9 \| 19,3 \| 19,2 \| \| Gross energy [MJ/kg] \| 16,3 \| n/a \| 22,5 \| 22,1 \| \| Fat [kJ%] \| 9,0 \| 60,0 \| 45,0 \| 43,0 \| \| Protein [kJ%] \| 33,0 \| 16,0 \| 20,0 \| 15,0 \| \| Carbohydrates [kJ%] \| 58,0 \| 24,0 \| 35,0 \| 42,0 \| \| crude ingredients \| \| \| \| \| \| Fat [%] \| 3,3 \| 35,1 \| 23,6 \| 22,0 \| \| Protein *(N x 6,25) [%] \| 19,0 \| 20,8 \| *22,0 \| *17,3 \| \| Fibre [%] \| 4,9 \| 0,4 \| 5,7 \| 5,6 \| \| Ash [%] \| 6,4 \| 3,1 \| 5,3 \| 4,2 \| \| N free extracts [%] \| 54,1 \| n/a \| 400,0 \| 48,3 \| \| Starch [%] \| 36,5 \| n/a \| 6,8 \| 4,8 \| \| Sugar (sucrose) [%] \| 4,7 \| 12,1 \| 21,1 \| 34,4 \| \| further specifications according to the manufacturer’s datasheets \| \| \| \| \| \|  \| **SD** \| **HFD** \| **DIO** \| **WSD** \| \| Feed composition \| grain and grain by-products, oil seed products, minerals, vegetable oils, vitamins, trace elements \| high fat (pork lard + palm oil) \| high fat (lard + corn oil), high sucrose, casein, minerals, vitamins, trace elements, choline chloride \| high fat (butter fat), high sucrose, casein, minerals, vitamins, trace elements, choline chloride \| \| Fat sources \| \| \| \| \| \| Soybean oil [%] \| n/a \| - \| 2,8 \| - \| \| Butter fat [%] \| n/a \| - \| - \| 21,0 \| \| Corn oil [%] \| n/a \| - \| - \| 1,0 \| \| Pork lard [%] \| n/a \| 30,5 \| 20,8 \| - \| \| Palm oil [%] \| n/a \| 4,5 \| - \| - \| \|  \| \| \| \| \| \|  \| **[%]** \| **[mg/kg]** \| **[%]** \| **[%]** \| \| Dextrine \| n/a \| n/a \| 11,0 \| 8,0 \| \| Polysaccharides \| n/a \| 148290,0 \| n/a \| n/a \| \| Corn starch \| n/a \| n/a \| 7,0 \| 5,0 \| \| Sucrose \| n/a \| n/a \| 20,2 \| 33,5 \| \| Monosaccharides \| n/a \| 28500,0 \| n/a \| n/a \| \| Dissacharides \| n/a \| 92330,5 \| n/a \| n/a \| \| Cellulose powder \| n/a \| n/a \| 5,7 \| 6,0 \| \| Cholesterol \| n/a \| n/a^$$^ \| n/a^$$^ \| 0,16 \|   ^$$^= contains undefined amounts of cholesterol from pork lard   \| Fatty acids \|  \| SD \| HFD \| DIO \| WSD \| \| --- \| --- \| --- \| --- \| --- \| --- \| \| Common name \| **Lipid numbers** \| **[%]** \| **[mg/kg]** \| **[%]** \| **[%]** \| \| Butanoic acid \| C 4:0 \| - \| - \| - \| 0,80 \| \| [Caproic acid](https://www.linguee.de/englisch-deutsch/uebersetzung/caproic+acid.html) \| C 6:0 \| - \| - \| - \| 0,53 \| \| Caprylic acid \| C 8:0 \| - \| - \| - \| 0,29 \| \| Capric acid \| C 10:0 \| - \| 300,00 \| - \| 0,63 \| \| Lauric acid \| C 12:0 \| - \| 300,00 \| 0,05 \| 0,72 \| \| Myristic acid \| C 14:0 \| 0,01 \| 5700,00 \| 0,29 \| 2,22 \| \| Palmitic acid \| C 16:0 \| 0,47 \| 93800,00 \| 5,33 \| 5,71 \| \| Margaric acid \| C 17:0 \| - \| 1300,00 \| - \| 0,14 \| \| Stearic acid \| C 18:0 \| 0,08 \| 63000,00 \| 2,92 \| 2,07 \| \| Arachidic acid \| C 20:0 \| 0,01 \| 1000,00 \| 0,07 \| 0,04 \| \| Palmitoleic acid \| C 16:1 \| 0,01 \| 7200,00 \| 0,62 \| 0,38 \| \| Oleic acid \| C 18:1 \| 0,62 \| 127900,00 \| 9,42 \| 4,90 \| \| [Linoleic acid](https://www.linguee.de/englisch-deutsch/uebersetzung/linoleic+acid.html) \| C 18:2 \| 1,80 \| 37400,00 \| 3,46 \| 0,93 \| \| Alpha-linolenic acid \| C 18:3 \| 0,23 \| 3250,00 \| 0,37 \| 0,12 \| \| Eicosanoic acid \| C 20:1 \| 0,02 \| 3900,00 \| - \| - \| \| Eicosadienoic acid \| C 20:2 \| - \| 1500,00 \| - \| - \| \| Arachidonic acid \| C 20:4 \| - \| 720,00 \| - \| - \| |
| --- | --- | --- | --- | --- | --- | --- | --- | --- | --- | --- | --- | --- | --- | --- | --- | --- | --- | --- | --- | --- | --- | --- | --- | --- | --- | --- | --- | --- | --- | --- | --- | --- | --- | --- | --- | --- | --- | --- | --- | --- | --- | --- | --- | --- | --- | --- | --- | --- | --- | --- | --- | --- | --- | --- | --- | --- | --- | --- | --- | --- | --- | --- | --- | --- | --- | --- | --- | --- | --- | --- | --- | --- | --- | --- | --- | --- | --- | --- | --- | --- | --- | --- | --- | --- | --- | --- | --- | --- | --- | --- | --- | --- | --- | --- | --- | --- | --- | --- | --- | --- | --- | --- | --- | --- | --- | --- | --- | --- | --- | --- | --- | --- | --- | --- | --- | --- | --- | --- | --- | --- | --- | --- | --- | --- | --- | --- | --- | --- | --- | --- | --- | --- | --- | --- | --- | --- | --- | --- | --- | --- | --- | --- | --- | --- | --- | --- | --- | --- | --- | --- | --- | --- | --- | --- | --- | --- | --- | --- | --- | --- | --- | --- | --- | --- | --- | --- | --- | --- | --- | --- | --- | --- | --- | --- | --- | --- | --- | --- | --- | --- | --- | --- | --- | --- | --- | --- | --- | --- | --- | --- | --- | --- | --- | --- | --- | --- | --- | --- | --- | --- | --- | --- | --- | --- | --- | --- | --- | --- | --- | --- | --- | --- | --- | --- | --- | --- | --- | --- | --- | --- | --- | --- | --- | --- | --- | --- | --- | --- | --- | --- | --- | --- | --- | --- | --- | --- | --- | --- | --- | --- | --- | --- | --- | --- | --- | --- | --- | --- | --- | --- | --- | --- | --- | --- | --- | --- | --- | --- | --- | --- | --- | --- | --- | --- | --- | --- | --- | --- | --- | --- | --- | --- | --- | --- | --- | --- | --- | --- | --- | --- | --- | --- | --- | --- | --- | --- | --- | --- | --- | --- | --- | --- | --- | --- | --- | --- | --- | --- |

Additional figure 1


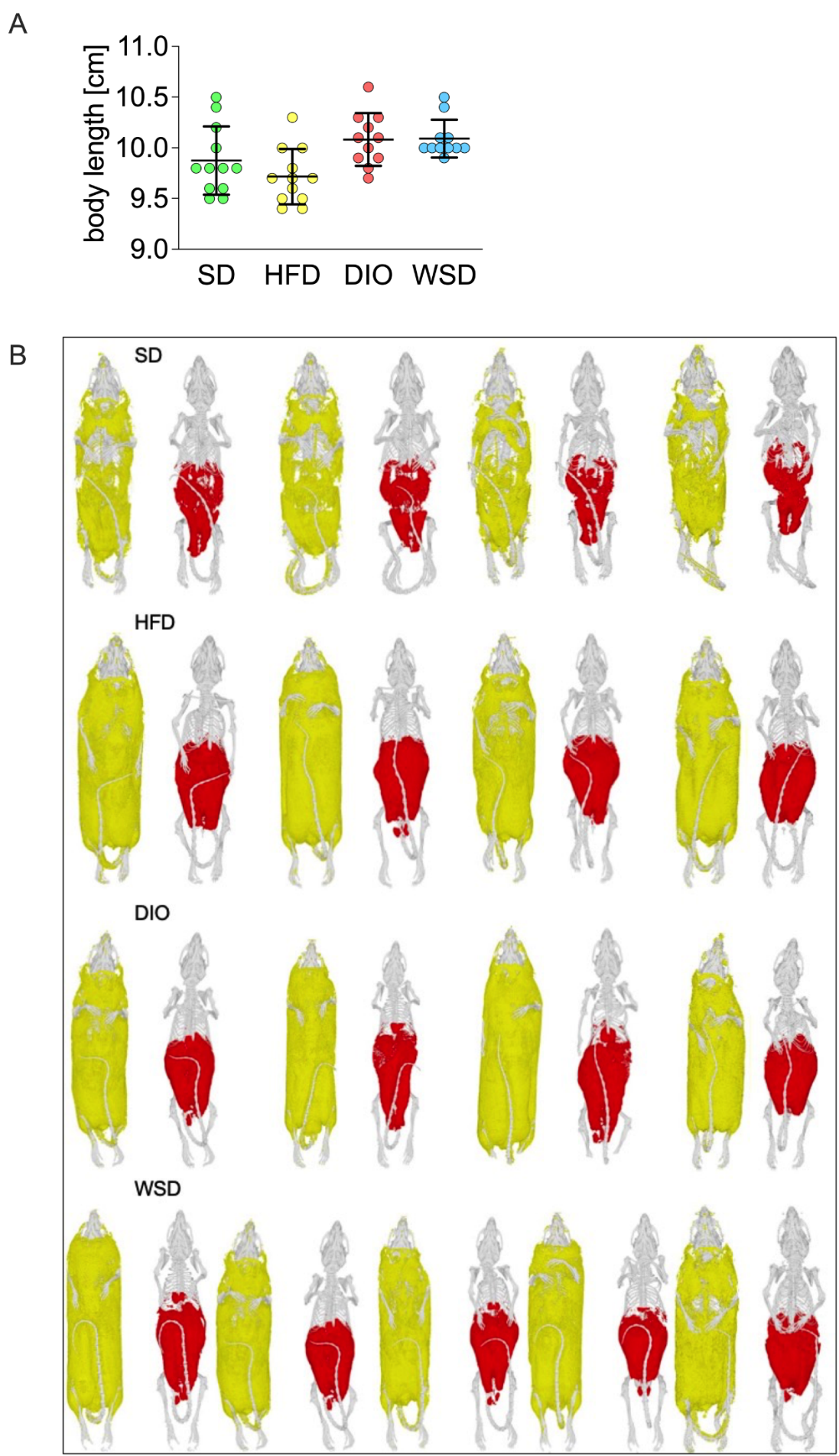


AF1: phenotypic results

A, body length, n= 11-12; B, µCT analyses: yellow = total fat; red = visceral fat.

Additional figure 2:


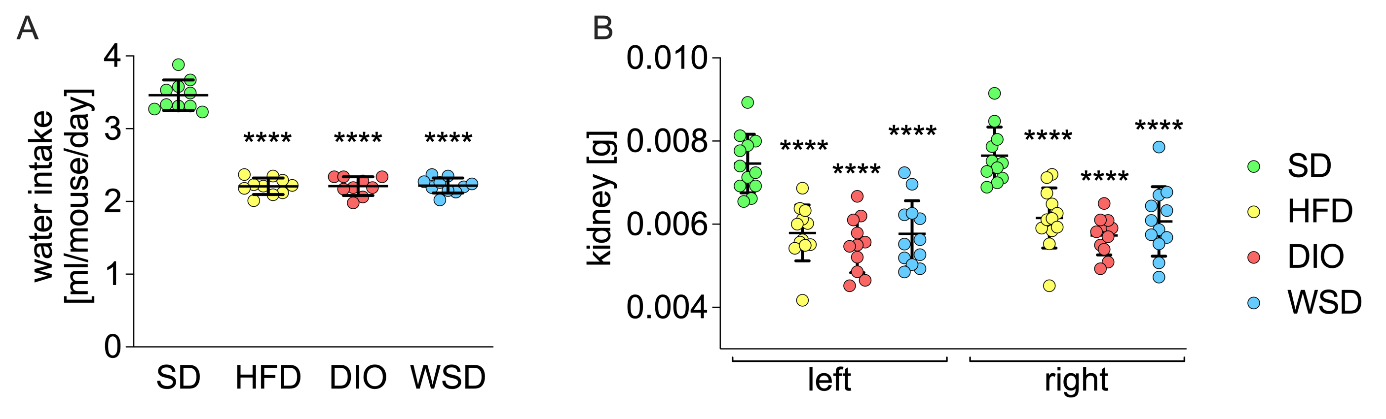


AF2: daily water intake during week 8-10 and kidney weights at P100

A, water intake, n = 9-10; B, kidney weights, n = 11-12.

Additional figure 3:

**
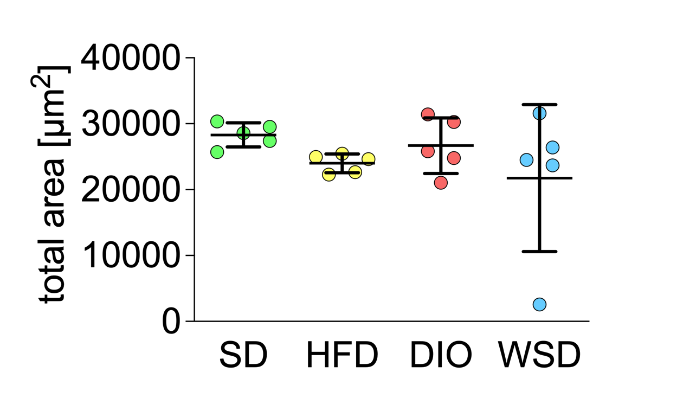
**

AF3: total area of measured adipocytes, n=5

Additional figure 4:


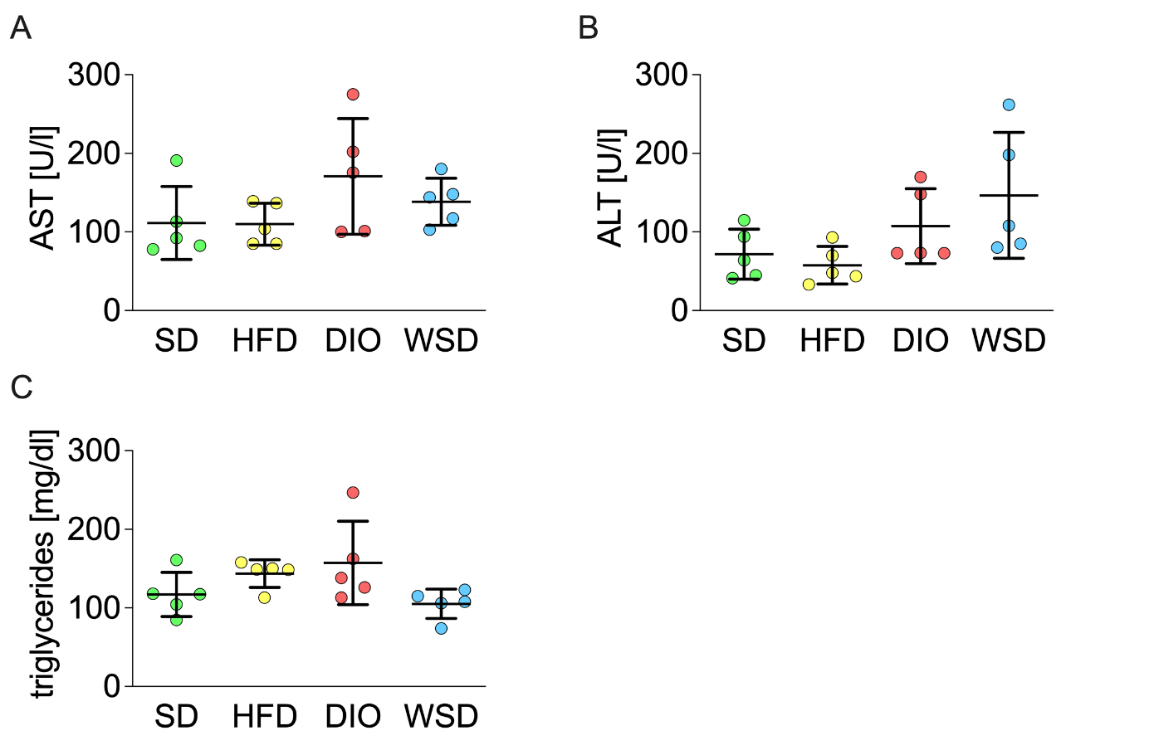


AF4: hepatic serum parameters

A, serum aspartate-aminotransferase (AST); B, serum alanine-aminotransferase (ALT); C, serum triglycerides; n=5.

AT2: expert examination of the H&E-stained liver sections

| sample | group | fat/inflammation | hydrops |
| --- | --- | --- | --- |
| 3 | SD | - | - |
| 32 | SD | - | - |
| 33 | SD | - | - |
| 65 | SD | - | - |
| 66 | SD | - | - |
| 17 | HFD | - | - |
| 44 | HFD | - | - |
| 45 | HFD | - | - |
| 48 | HFD | fat focally 5% | - |
| 78 | HFD | - | - |
| 23 | DIO | fat 5% | + |
| 50 | DIO | fat 30% = NASH | + |
| 54 | DIO | slight inflammation | n/a |
| 80 | DIO | fat 5% | + |
| 83 | DIO | n/a | + |
| 27 | WSD | n/a | + |
| 60 | WSD | n/a | + |
| 86 | WSD | fat 15% | + |
| 87 | WSD | fat 15% | +++ |

AT3: IC cluster export (see additional (excel) file 2)

AT4: Venn data (see additional (excel) file 3)
